# Supplementary material for: Hepatic transcriptomic landscape of chicken reveals host responses to spotty liver disease
Source: PLoS One. 2026 Sep 16;21(9):e0346466. doi: 10.1371/journal.pone.0346466 (PMC13580954; doi:10.1371/journal.pone.0346466)
Supplement: Supplementary file 1 [file pone.0346466.s001.pdf]

FBP2 FP: GGTGACGGAGGAGAACAAGG

FBP2 RP: TTCCTATGGGTGCCAAGCAG

RAD51C FP: AACGCAGGGCTTCATCATCA

RAD51C RP: TTTTCCCAACACCAGGTGCT

IL-6FP AGGACGAGATGTGCAAGAAGTTC

IL-6RP TTGGGCAGGTTGAGGTTGTT

IL- 22FP TGTTGTTGCTGTTTCCCTCTTC

IL- 22RP CACCCCTGTCCCTTTTGGA
