# Supplementary material for: Hepatic transcriptomic landscape of chicken reveals host responses to spotty liver disease
Source: PLoS One. 2026 Sep 16;21(9):e0346466. doi: 10.1371/journal.pone.0346466 (PMC13580954; doi:10.1371/journal.pone.0346466)
Supplement: Supplementary file 2 [file pone.0346466.s005.pdf]

Downregulated Immune Genes

Gene	Log fold change	P value	FDR -P Value
LOC121106526	-7.286278069	3.62E-04	8.23E-04
LOC121107862	-5.142209099	0.010629088	0.019612893
LOC112531339	-5.26578175	8.41E-03	0.015768462
LOC107050230	-6.61367768	6.44E-04	1.42E-03

Downregulated Metabolic genes

Gene	Log fold change	P value	FDR -P Value
FBP2	-7.277014258	1.5E-04	3.54E-04
HKDC1	-5.032875164	0	0
ALDOB	-2.432264742	5.47E-267	2.3E-264
PDHA2	-2.084667426	2.35E-187	5.09E-185
GLUL	-2.047754898	5.33E-183	5.33E-183
APOB	-2.013155611	8.31E-189	1.82E-186
APOA2	-2.601708299	1.28E-276	5.98E-274
PLIN1	-4.356851228	3.99E-30	3.72E-29
OTC	-5.911446451	1.98E-44	2.95E-43
PYCR1	-3.64365771	1.03E-292	5.69E-290
CYP2C18	-3.512294834	0	0
CYP2AC1	-3.228046645	0	0
ACAA1	-2.454879821	2.3E-26	9.51E-263

SLC30A2	-5.157163646	1.52E-15	7.64E-15
C1QTNF1	12.34271058	4.93E-04	1.1E-03
ADGRD1	11.70120881	9.53E-04	2.05E-03
